# Evaluation of the antimicrobial and NorA and MepA efflux pump inhibitory activity of a hydrazone derivative of hydralazine against *Staphylococcus aureus*

**DOI:** 10.1007/s00203-026-05007-0

**Published:** 2026-06-19

**Authors:** Karla Susanna Tavares Grangeiro Belém, Janaina Esmeraldo Rocha, Ana Joyce Morais Bento, Jaiza Maria Lima Dias, Henrique Douglas Melo Coutinho, Francisco das Chagas Lima Pinto, Ramon Róseo Paula Pessoa Bezerra de Menezes, Mateus Edson da Silva, Emmanuel Silva Marinho, Marcia Machado Marinho, Hélcio Silva dos Santos

**Affiliations:** 1 Graduate Program in Biological Chemistry, Department of Biological Chemistry, Regional University of Cariri, Crato, CE Brazil; 2 Chemistry Course, Vale do Acaraú State University, Sobral, Ceará Brazil; 3 Graduate Program in Natural Sciences, State University of Ceará, Fortaleza, CE Brazil; 4 Graduate Program in Chemistry, Federal University of Sergipe, São Cristovão, SE Brazil; 5 Department of Clinical and Toxicological Analysis, Federal University of Ceará, Fortaleza, CE Brazil

**Keywords:** Bacterial resistance, Efflux pumps, NorA, MepA, Hydrazone

## Abstract

Bacterial resistance represents one of the major challenges in contemporary public health, compromising the effectiveness of conventional antibiotics. In this context, research focused on the discovery of new compounds and resistance modulators has gained increasing attention. The present study aimed to evaluate the antimicrobial activity and the antibiotic-modulating potential of a hydrazone derivative of hydralazine against *Staphylococcus aureus* strains harboring efflux pumps. The compound (*E*)-2-((2-(phthalazin-1-yl)hydrazineylidene)methyl)phenol (HDZH2OH) was synthesized from hydralazine and isoniazid and evaluated using the Minimum Inhibitory Concentration (MIC) assay by the broth microdilution method, as well as resistance modulation assays in association with specific antibiotics and ethidium bromide. The cell viability assay showed that HDZH2OH reduced macrophage viability in a concentration-dependent manner, with viability decreasing from 74.8 ± 3.0% at 3.25 µM to 27.25 ± 1.25% at 25 µM. MIC values ≥ 1024 µg/mL (3.88 mM) against both tested strains, indicating the absence of intrinsic antibacterial activity. However, in the *S. aureus* 1199B strain, a reduction in the MIC of norfloxacin from 256 µg/mL to 101.59 µg/mL (0.802 mM to 0.334 mM) was observed, suggesting a partial potentiating effect. In the K2068 strain, no potentiation of ciprofloxacin was observed; however, a significant reduction in the MIC of ethidium bromide from 8 µg/mL to 2 µg/mL (0.020 mM to 0.005 mM) was detected (*p* < 0.001). Molecular docking indicated that HDZH2OH strongly interacts with *S. aureus* efflux pumps, showing high affinity and selective binding. It overlaps with substrate sites in MepA and NorA, suggesting competitive inhibition. Its interactions, including hydrogen bonds, enhance stability, highlighting its potential to inhibit efflux activity and restore antimicrobial effectiveness. HDZH2OH shows favorable drug likeness consistent with Lipinski and Veber rules. It is mostly neutral at physiological pH (pKa 4.05), supporting good permeability and oral absorption. CNS MPO score (4.47) suggests potential CNS activity and BBB penetration. It has high plasma protein binding and moderate toxicity risk, with possible CYP inhibition and hepatotoxicity concerns. These findings indicate that the compound acts as a selective efflux pump modulator, with greater affinity for the MepA system, highlighting its potential as a therapeutic adjuvant in combating bacterial resistance.

**Graphical abstract:**

**Figure:**
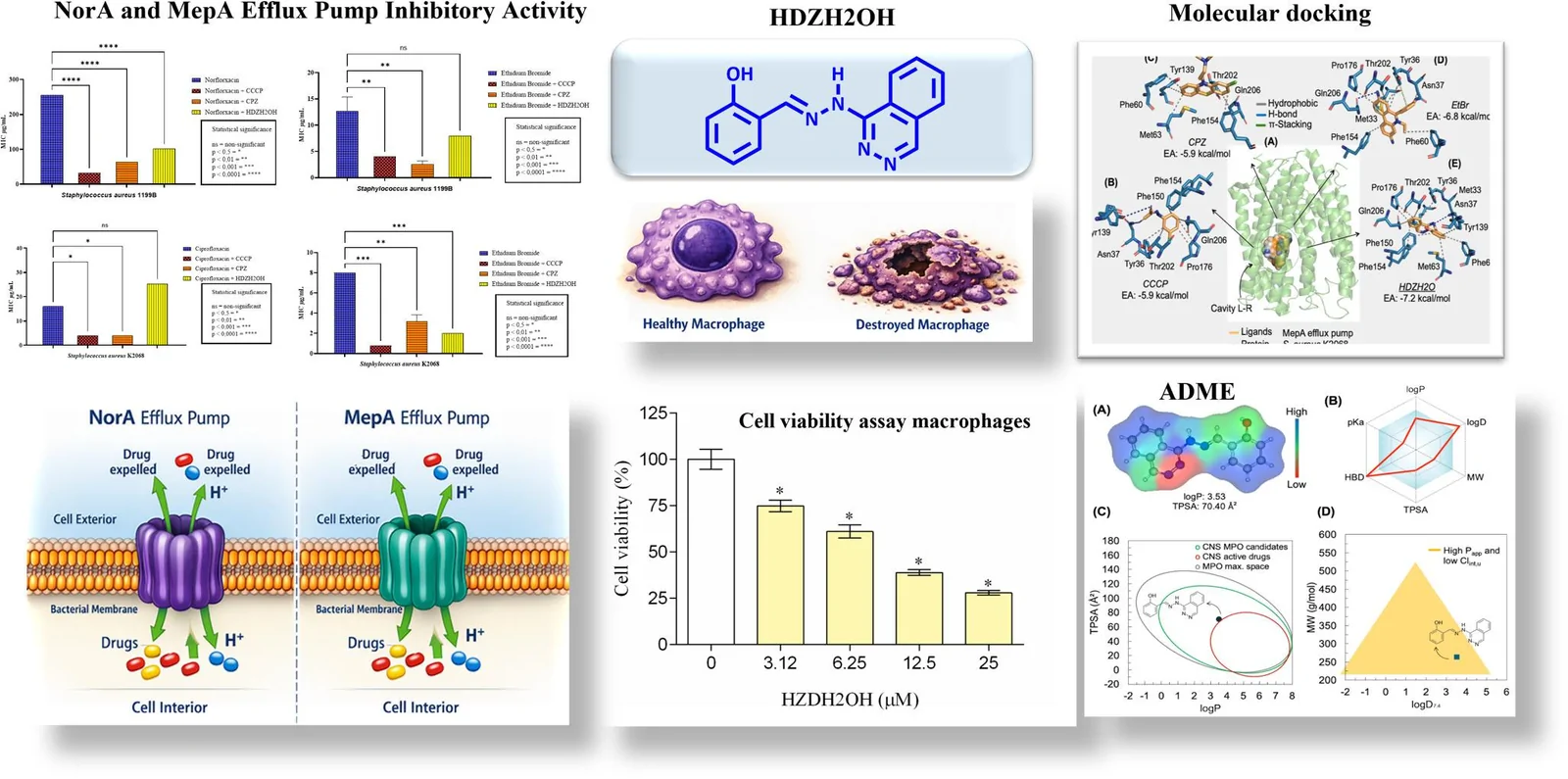

**Supplementary Information:**

The online version contains supplementary material available at https://doi.org/10.1007/s00203-026-05007-0.

## Introduction

Bacterial resistance is a major global public health concern, as it reduces the effectiveness of antibiotics and compromises the treatment of infections that were once easily managed with conventional antimicrobial agents (Patini et al. 2020), leading to treatment failure, increased transmission of severe diseases, and higher morbidity and mortality rates (Murugaiyan et al. 2022). Among resistant pathogens, *Staphylococcus aureus* is one of the most clinically relevant multidrug-resistant bacteria due to its strong ability to acquire diverse resistance mechanisms, being associated with severe infections that may rapidly progress and result in life-threatening outcomes (Walvekar et al. 2019). This species has developed resistance to several antibiotic classes used against Gram-positive infections, including β-lactams, glycopeptides, and oxazolidinones (Guo et al. 2020; Mlynarczyk-Bonikowska et al. 2022).

Given the problems caused by the development of antibiotic resistance, new therapeutic alternatives have been actively sought (Nunes et al. 2022), and the synthesis of novel molecules stands out as an excellent strategy in the search for new antibacterial agents, as it enables the rational design and structural modification of compounds, allowing the optimization of biological activity and selectivity, as well as the exploration of new chemical spaces to overcome existing resistance mechanisms (Akın et al. 2026; Kundu et al. 2025; Jamshidi et al. 2025; Farzandi et al. 2025).

Among these, hydralazine is a well-known vasodilator widely used in the treatment of hypertension and heart failur demonstrated additional pharmacological potential (Romão et al. 2025), including antifungal activity against Candida spp. and antibacterial effects against methicillin-resistant *S. aureus* (Nascimento et al. 2023, 2024). In this context, hydrazone derivatives from hydralazine might constitute an important class of bioactive molecules in pharmaceutical chemistry, with their biological activity largely associated with the presence of the azomethine pharmacophore (Feizi-Dehnayebi et al. 2025a, b; Alves et al. 2025). Importantly, these intrinsic antimicrobial properties of hydralazine and hydrazone moiety have motivated its use as a structural scaffold for the development of more active derivatives (Moreira et al. 2026; Nery et al. 2023). Therefore, the present study aims to evaluate a hydrazone (*E*)-2-(2-(phthalazin-1-yl)hydrazineylidene)methyl)phenol (HDZH2OH) derivative of hydralazine, focusing on its antimicrobial activity and efflux pump inhibition capacity in *S. aureus* strains.

## Materials and methods

### Synthesis

**Scheme 1:**
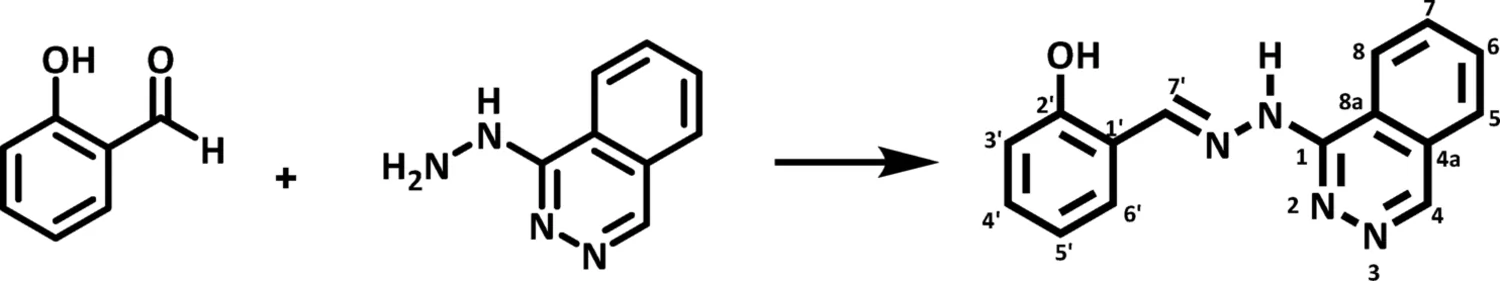
Synthesis of hydrazone. ^1^H NMR (500 MHz, DMSO); *δ*_H_ 12.16 (s, NH-1), 10.33 (s, OH-2’), 8.69 (s, H-7”), 8.31 (d, *J* = 7.7 Hz, H-8), 8.11 (s, H-4), 7.84 (d, *J* = 7.5 Hz, H-3’), 7.75 (3 H, m, H-5/H-6/H-7), 7.27 (t, *J* = 7.6 Hz, H-4’), 6.92 (d, *J* = 7.5 Hz, H-6’), 6.89 (t, *J* = 7.6 Hz, H-5’). ^13^C NMR (125 MHz, DMSO); *δ*_H_ 157.0 (C-2’), 154.0 (C-7’), 147.0 (C-1), 137.9 (C-4), 132.2 (C-6), 131.8 (C-7), 131.0 (C-4’), 130.0 (C-1’), 126.9 (C-4a), 126.4 (C-5), 126.1 (C-8a), 123.7 (C-8), 120.2 (C-6’), 119.2 (C-5’), 116.1 (C-3’)

In a 25 mL round-bottom flask, 0.50 mmol of 2-hydroxybenzaldehyde was mixed with 0.50 mmol of 1-hydrazineylphthalazine (hydralazine), 9.0 mL of distilled water, and 1.0 mL of concentrated H₃PO₄ (Scheme 1). The reaction mixture was stirred magnetically and heated at 100 °C for 45 min. Subsequently, 15 mL of absolute ethanol was added to the reaction mixture, which was then filtered, and the filtrate was collected in a beaker. The solid residue retained on the filter paper was discarded. Next, 20 mL of a cold 5.0% (w/v) aqueous NaHCO₃ solution was added to the filtrate. The resulting solid was collected by vacuum filtration, washed with cold absolute ethanol, and dried at room temperature. Finally, the product was removed from the filter paper, weighed, and characterized. The structure of the hydrazone was confirmed by spectroscopic analysis, yielding 60% with a purity of 99.69% (Figure S3, supplementary material) and melting point ranging from 198 to 200 °C, in agreement with the value reported (Arruda et al. 2020).

### Spectroscopic methods

^1^H and ^13^C NMR spectra were obtained using Bruker DRX 500 MHz, operating at a frequency of 500 MHz for hydrogen, and 125 MHz for carbon, respectively. The spectra were measured in DMSO-d6 solvent, and chemical shifts are reported as δ values in parts per million (ppm). Analyses were performed with a Shimadzu Prominence Auto Sampler (SIL-20 A) HPLC system (Shimadzu, Kyoto, Japan) equipped with Shimadzu LC-20AD reciprocating pumps connected to a DGU 20A5 degasser with a 20 A integrating CBM, SPD-M20A diode array detector, and LC Solution 1.22 SP1 software. Shim-pack (CLC) ODS GOLD (4.6 × 250 mm, 5 μm) reversed-phase column coupled to a Shim-pack (CLC) G-ODS 4 reversed-phase guard column (4.0 × 10 mm, 5 μm). Mobile phase: HPLC-grade methanol. Injection volume: 20µL. Flow rate: 0.5 mL/min. Elution mode: Isocratic. Reading wavelength: 350 nm.

### Substances

Efflux pump inhibitors used as controls were carbonyl cyanide m-chlorophenylhydrazone (CCCP) and chlorpromazine (CPZ). The antibiotics specific to each efflux pump were norfloxacin for the NorA pump in strain 1199B and ciprofloxacin for the MepA pump. From all substances, stock solutions (1024 µg/ml) were prepared, by dissolving 10 mg of each substance in 500 µL of dimethyl sulfoxide (DMSO) and sterile distilled water (9265 µL). Ethidium bromide was also used, diluted only in distilled water (9765 µL). All reagents were obtained from Sigma-Aldrich Co. (St. Louis, MO, USA). The culture media employed included Heart Infusion Agar (HIA), Brain Heart Infusion Broth (BHI) broth prepared at 10% (w/v), blood agar, and glycerol, all purchased from Difco Laboratories Ltd.

### Microorganisms used in the inhibition assays

The *Staphylococcus aureus* strains used were 1199B and K2068, which are resistant to hydrophilic fluoroquinolones via the NorA and MepA efflux proteins, respectively. All strains were obtained from the Microbiology and Molecular Biology Laboratory (LMBM) of the Regional University of Cariri (URCA).

### Preparation and standardization of inocula

For the Minimum Inhibitory Concentration (MIC) assays, bacterial strains re-trieved from frozen stocks stored at -20 °C were subcultured on Heart Infusion Agar (HIA) plates and incubated at 37 °C for 24 h. Subsequently, a few well-isolated colo-nies were suspended in sterile saline solution, and the turbidity was adjusted to the 0.5 McFarland scale, corresponding to approximately 10⁸ CFU (colony-forming units). This standardized inoculum was used for both MIC determination and efflux pump inhibi-tion assays.

### Minimum inhibitory concentration assays

MIC values were determined by the broth microdilution method in 96-well plates. From solid HIA stock cultures, bacteria were subcultured onto HIA plates and incubated at 37 °C for 24 h. Subsequently, microtubes were filled with 100 µL of inoculum and 900 µL of liquid BHI medium. The contents of the microtubes were transferred to 96-well microdilution plates, with 100 µL added to each well. Serial microdilutions of the test substances were then performed by adding 100 µL to the first well of each column. Two-fold serial dilutions were performed across the wells up to the penultimate well. The last well served as growth control. Concentrations ranged from 512 µg/mL to 8 µg/mL (1.94 mM to 0.03 mM), following the standard MIC determination methodology (Kadeřábková et al. 2024). Sterility control consisted of wells containing only BHI Broth, whereas growth controls consisted of inoculated wells without test compounds.After 24 h of incubation, 20 µL of resazurin (7-hydroxy-3 H-phenoxazin-3-one 10-oxide) were added to each well, and the plates were incubated for an additional 1 h before visual inspection based on color change. Resazurin reduction by metabolically active cells results in a color change from blue to pink/red, indicating bacterial growth, whereas persistence of the blue color indicates growth inhibition (Herman et al. 2024). MIC was defined as the lowest concentration at which no color change was observed (blue), indicating absence of visible bacterial growth. MIC values of all experiments are presented as the mean ± standard deviation from at least three independent experiments, each performed in triplicate, confirming reproducibility of the reported inhibitory limits.

### Efflux reduction assay by MIC decrease

To assess the reduction of antibiotics and ethidium bromide MIC, strains were cultured and inocula prepared as described above. Test and control distribution media were prepared in microtubes. For the test group, 150 µL of inoculum and volume corresponding to the subinhibitory concentration (MIC/8) of the test compound were added, and the final volume was adjusted to 1.5 mL, as well as the CCCP and CPZ. The control contained the same inoculum volume, adjusted to the same final volume by addition of BHI (1350 µL).The contents of these microtubes were transferred to 96-well microdilution plates in a vertical distribution, with 100 µL added to each well. Serial microdilutions of antibiotics - norfloxacin for NorA and ciprofloxacin for MepA - and ethidium bromide were performed (1:1), with concentrations ranging from 512 µg/mL to 0.5 µg/mL. (1.3 mM to 0.001 mM), until the penultimate well, as done in the MIC assay. After 24 h of incubation, plates were visually inspected to determine MIC endpoints using resazurin as described above. A reduction in the MIC of ethidium bromide or specific antibiotics in efflux pump–harboring strains was considered indicative of efflux system inhibition, according to Tintino et al. (2020). Sterility and bacterial growth control were performed as described above. All experiments are presented as the mean ± standard deviation from at least three independent experiments, each performed in triplicate, confirming reproducibility of the reported inhibitory limits.

### Citotoxicity assay

The murine macrophage cell line RAW264.7 (ATCC TIB-71), obtained from the Rio de Janeiro Cell Bank (BCRJ, Brazil), was employed to evaluate the cytotoxicity of HDZH2OH using the MTT assay (Mosmann 1983). Cells were cultured in Dulbecco’s Modified Eagle Medium (DMEM) supplemented with 10% fetal bovine serum (FBS), 100 U/mL penicillin, and 100 µg/mL streptomycin, and maintained at 37 °C in a humidified atmosphere containing 5% CO₂. A suspension of 1 × 10⁵ cells/mL was seeded into sterile 96-well plates and incubated overnight to allow adhesion and proliferation. Cells were subsequently treated with different concentrations of HDZH2OH for 24 h. After treatment, wells were washed with phosphate-buffered saline (PBS) and incubated with MTT solution (0.25 mg/mL) for 4 h at 37 °C. The reduction of MTT by mitochondrial dehydrogenases resulted in the formation of purple formazan crystals, serving as the colorimetric indicator of metabolically active cells. These crystals were solubilized in dimethyl sulfoxide (DMSO), and absorbance was measured spectrophotometrically at 570 nm using a microplate reader. Cell viability was expressed as a percentage relative to the untreated control group, and as a negative control, cells exposed only to vehicle (0.5% DMSO) were included to account for solvent effects.

### Statistical analysis

Results were expressed as geometric mean ± standard deviation because MIC values are derived from serial two-fold dilution data. Central values and standard deviations were obtained following the methodology described by Freitas et al. (2021) for microbiological analysis in microdilution plates. Data were analyzed using one-way ANOVA followed by Bonferroni’s post hoc test for pairwise comparisons among control and treatment groups, using GraphPad Prism 6.01. Values of *p* < 0.05 were considered statistically significant.

### Molecular docking

The HDZH2OH structure was constructed using MarvinSketch (ChemAxon) and subsequently subjected to conformational optimization. Energy minimization was performed using Avogadro (Snyder & Kucukkal, 2017), employing the MMFF94 force field (Halgren, 1996) and the steepest descent algorithm (Donchev et al. 2005), with 500 iterations and a convergence criterion of 1 × 10⁻⁷. The amino acid sequences of MepA from *S. aureus* K2068 and NorA from *S. aureus* 1199B were adapted according to De Araújo et al. (2025) and Freitas et al. (2024, 2022). The MepA sequence was retrieved from the UniProt database under accession NCTC8325 (Pundir et al. 2016; Warraich et al. 2021). Protein modeling was performed using the SWISS-MODEL server, with template identification carried out using the HHblits algorithm (Remmert et al. 2012). The MATE transporter from *Bacillus halodurans* (PDB: 5C6N) was selected as the structural template based on sequence similarity (Costa et al. 2025; Radchenko et al. 2015). The NorA structure was obtained from the RCSB Protein Data Bank (PDB: 7LO7), corresponding to a NorA–Fab25 complex with a resolution of 3.74 Å, determined by cryo-electron microscopy and previously characterized as a transport protein from *S. aureus*/*Homo sapiens* (Brawley et al. 2022).

Molecular docking simulations were performed using AutoDock Vina (Trott and Olson 2010), employing grid boxes configured to fully encompass the target structures. For the MepA protein, the grid box was centered at coordinates 9.189 (x), − 19.589 (y), and − 19.523 (z), with dimensions of 84 (x), 98 (y), and 118 Å. For NorA, the grid box was centered at 139.133 (x), 138.065 (y), and 155.834 (z), with dimensions of 108 (x), 126 (y), and 126 Å. To validate the docking protocol, redocking simulations were performed by re-docking co-crystallized ligands into their respective protein targets, using an exhaustiveness parameter of 64 to ensure robust sampling of the conformational space. Validation was assessed by calculating the root-mean-square deviation (RMSD) between predicted and experimental poses, with values ≤ 2.0 Å considered satisfactory (Yusuf et al. 2008). Binding affinity was used as an indicator of complex stability, with values lower than − 6.0 kcal/mol considered energetically favorable (Shityakov & Förster, 2014; Agarwal and Smith 2023). All simulations were conducted under identical parameters to ensure methodological consistency and enable direct comparison. The ligands were evaluated against the reference compounds carbonyl cyanide m-chlorophenyl hydrazone (CCCP), chlorpromazine (CPZ), and ethidium bromide (EtBr), used as positive controls.

### Drug-likeness and ADME properties

The calculated parameters were evaluated according to drug-likeness criteria, following the classical Lipinski’s rule of five (Lipinski 2004; Lipinski et al. 2012) and Veber’s rule (Veber, 2002), which define critical thresholds for properties such as logP, molecular weight, number of hydrogen bond donors and acceptors, topological polar surface area (TPSA), and number of rotatable bonds. Pharmacokinetic parameters related to absorption, distribution, metabolism, and excretion (ADME) were estimated using a consensus approach, combining predictive data from the PreADMET server, based on in vitro models, with results from in silico screening tools provided by SwissADME, thereby increasing the robustness of predictions regarding the compound’s biopharmaceutical properties. The identification of pharmacophoric groups was performed through high-throughput virtual screening (HTS) using the eMolTox platform, enabling the automated detection of substructures associated with toxicity, chemical reactivity, or metabolic instability based on predictive algorithms. Toxicological alerts were further analyzed using HDZH2OH as a structural similarity reference, and the analysis was complemented with predictions from PreADMET and ProTox-3.0, allowing the assessment of potential toxicological risks, including hepatotoxicity, mutagenicity, carcinogenicity, and acute toxicity.

## Results and discussion

In the ^1^H NMR and ^13^C spectrum of HDZH2OH (Figures S1 and S2) the signals at 7.84 (d, *J* = 7.5 Hz, H-3’), δ_H_ 7.27 (t, J = 7.6 Hz, H-4’), 6.89 (t, J = 7.6 Hz, H-5’), 6.92 (d, J = 7.5 Hz, H-6’) are attributed to the protons of the phenolic ring protons ring present in the molecule. These assignments are further supported by the corresponding carbon signals observed at δ_C_ 116.1 (C-3’), 131.0 (C-4’), 119.2 (C-5’), 120.2 (C-6’) in the ^13^C NMR spectrum. The signals at δ_H_ 8.11 (s, H-4), 7.75 (3 H, m, H-5/H-6/H-7) and 8.31 (d, *J* = 7.7 Hz, H-8) are attributed to the aromatic protons of the phthalazine ring, these assignments are further supported by the corresponding carbon signals observed at δ_C_ 137.9 (C-4), 126.4 (C-5), 132.2 (C-6), 131.8 (C-7) and 123.7 (C-8). In addition, singlets at *δ*_H_ 12.16 (s, NH-1), δ_C_ 154.0 (C-7’) and 8.69 (s, H-7’) were observed, corresponding to the N–H and the azo-methine protons (HC = N), respectively. The presence of the phenolic OH proton signal can be observed at δ_H_ 10.33 (s, OH-2’) and further confirmed by the signal at δ_C_ 157.0 (C-2’) of the carbon atom bonded to oxygen. The remaining signals correspond to non-protonated sp² carbons at δ_C_ 147.0 (C-1),130.0 (C-1’), 126.9 (C-4a), 126.1 (C-8a).

UV spectrum of HDZH2OH hydrazone (Figure S4) exhibited absorption maximum at 210 nm is attributed to π → π* transitions indicate the presence of aro-matic rings, confirmed by the band at 287 nm that showed a more extended conjugated aromatic system. The presence of an expanded π-conjugated system involving conjuga-tion between the benzene ring and the (C = N) moiety was confirmed by the band at 370 nm, which further contributes to the extended conjugation. The absorption peak at 370 nm confirms significant electronic coupling between the two aromatic systems. In summary, the analysis of the spectroscopic data, allowed the unequivocal determination of the hydrazone structure as HDZH2OH (Scheme 1). In addition, the purity of HDZH2OH evaluated by high-performance liquid chroma-tography (HPLC; Figure S4), was 99.69%, purity.

The compound HDZH2OH shows potential novelty mainly due to the uncommon combination of three distinct structural fragments a phthalazine heteroaromatic core, a Schiff base (hydrazone) linkage, and an ortho-hydroxybenzyl (salicylaldehyde-derived) phenolic unit within a single conjugated molecular framework. Notably, the phthalazine heteroaromatic core can be conceptually related to the well-known antihypertensive drug hydralazine, from which phthalazine based hydrazine chemistry is historically derived. While each of these motifs is individually well documented in organic and coordination chemistry, their specific integration into a single system is relatively rare. The resulting structure features extended π-conjugation (Feizi-Dehnayebi et al. 2025a, b). This structure exhibits a conjugated system capable of acting as both a hydrogen bond donor and acceptor, as well as moderate lipophilicity. These structural features favor interactions with bacterial membranes and transmembrane proteins, such as efflux pumps, without necessarily inducing a direct bactericidal effect. Previous studies indicate that hydrazones with this structural profile are more likely to modulate resistance mechanisms than to directly inhibit bacterial growth, which is consistent with the findings of this study (Rollas and Küçükgüzel 2007; Nery et al. 2023).

**Fig. 1:**
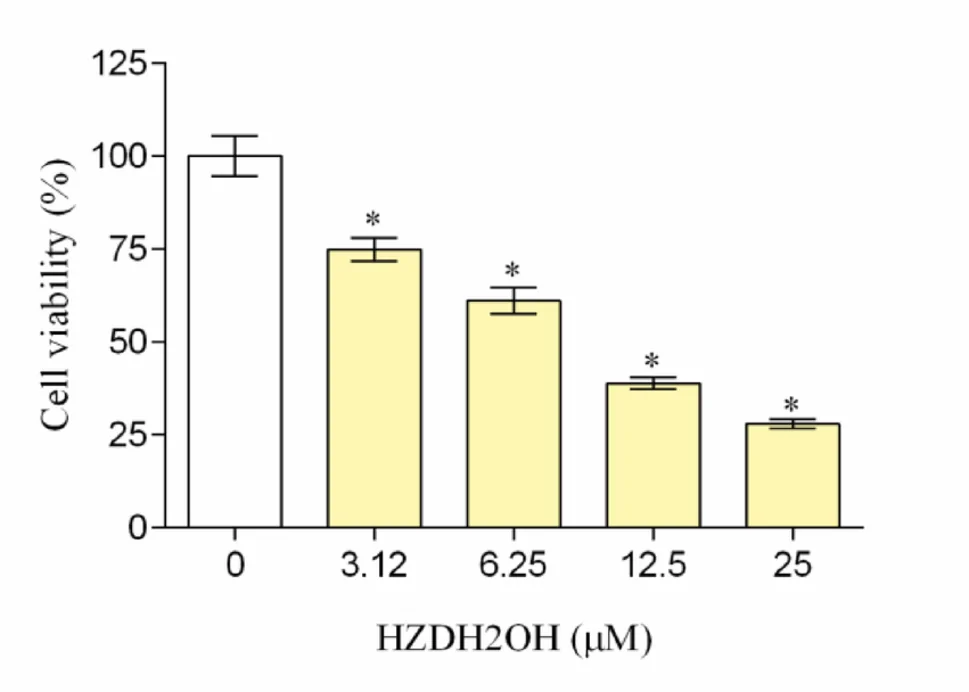
Cytotoxicity of HDZH2OH

The cell viability assay demonstrated that HDZH2OH exerts a concentration-dependent cytotoxic effect on macrophages, as evidenced by the progressive reduction in cell viability with increasing concentrations of the compound. At 3.12 µM, cell viability decreased to approximately 74.8%, indicating a moderate cytotoxic effect, while higher concentrations such as 6.25 µM further reduced viability to around 60%, demonstrating an intensification of toxicity. The strongest effects were observed at 12.5 and 25 µM, where viability decreased markedly to approximately 38% and 27.25%, respectively, confirming significant cytotoxic activity. Importantly, the calculated IC₅₀ value was 10.66 ± 2.37 µM, confirming the potency of HDZH2OH in reducing macrophage viability (Fig. 1).

In the present study, MIC values for HDZH2OH against *S. aureus* strains 1199B (NorA) and K2068 (MepA) were ≥ 1024 µg/mL (3.88 mM). Considering that compounds with relevant antibacterial potency typically display substantially lower MIC values, usually in the micromolar or low µg/mL range, these results suggest low intrinsic antibacterial activity under the tested conditions. However, limited direct antibacterial activity does not necessarily indicate pharmacological inactivity, since compounds that fail to inhibit growth when tested alone may still act as resistance modulators by sensitizing bacteria to conventional antibiotics. In the case of HDZH2OH, the elevated MIC may reflect limited membrane disruption capacity, insufficient intracellular accumulation, or preferential interaction with resistance-associated proteins rather than essential bacterial growth targets.

**Fig. 2:**
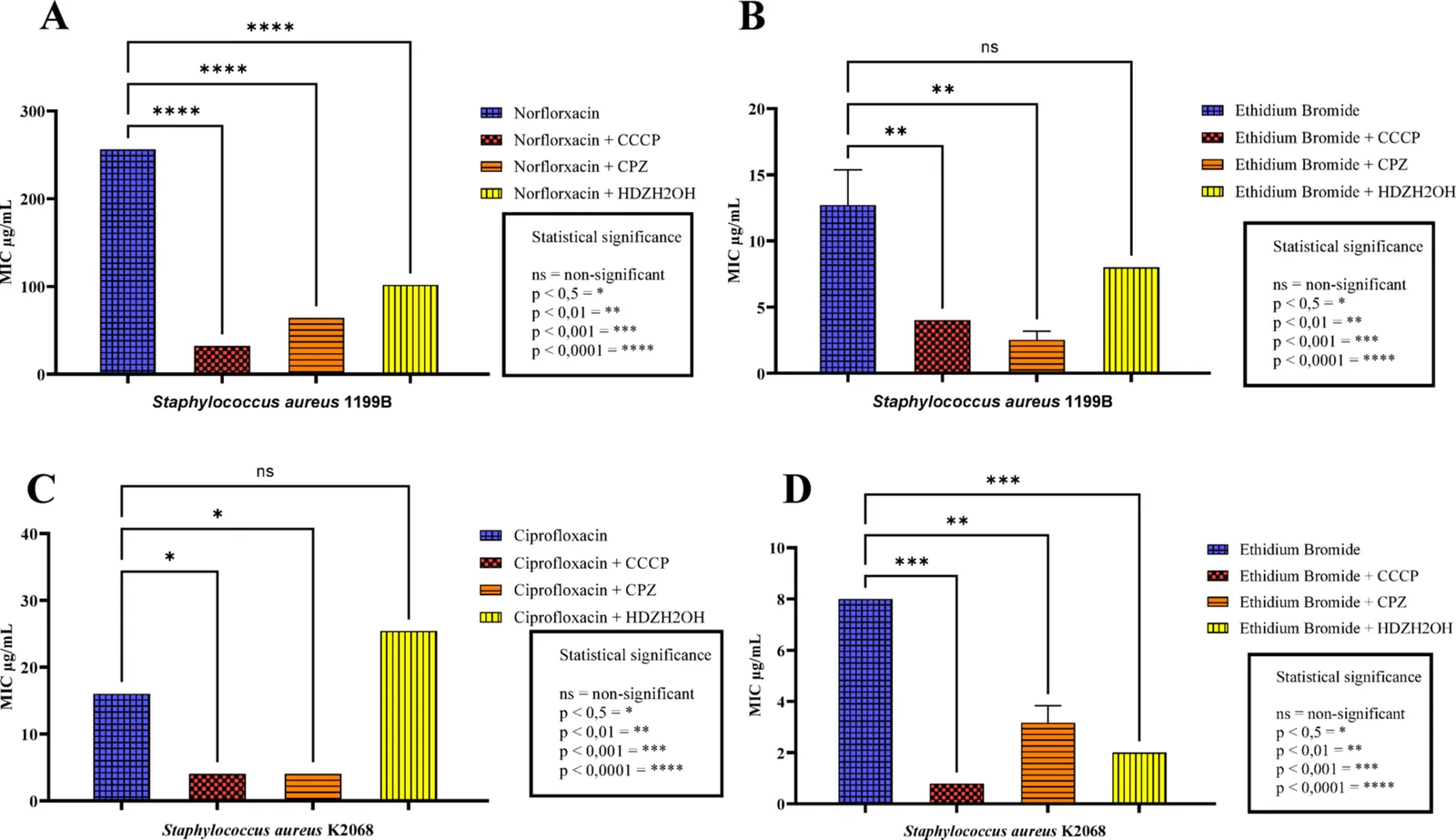
MICs of norfloxacin, ciprofloxacin, and ethidium bromide in the absence and presence of HDZH2OH, chlorpromazine (CPZ), and CCCP against *S. aureus* strains 1199B (NorA) and K2068 (MepA). MIC values are presented as the mean ± standard deviation from at least three independent experiments, each performed in triplicate, confirming reproducibility of the reported inhibitory limits

This behavior is consistent with several studies demonstrating that hydrazones and hydralazine derivatives often lack strong direct antimicrobial activity but primarily act as resistance modulators, interfering with bacterial adaptive mechanisms, particularly efflux pumps (Coutinho et al. 2011; Tintino et al. 2020; Nery et al. 2023). When associated with norfloxacin against *S. aureus* strain 1199B, a reduction in the MIC from 256 µg/mL to 101.59 µg/mL (0.802 mM to 0.334 mM) was observed, indicating a possible potentiating effect, as shown in Fig. 2A. However, this reduction was less pronounced than that observed in the presence of CCCP and CPZ, suggesting partial or indirect activity on the resistance mechanism.

Similar results were reported by Nascimento et al. (2024), who demonstrated that hydralazine and its derivatives can potentiate antibiotics against methicillin-resistant *S. aureus* without exhibiting significant antibacterial activity when tested alone. These findings support the hypothesis that hydrazone compounds may function as therapeutic adjuvants, partially restoring antibiotic efficacy compromised by efflux mechanisms.

Figure 2B shows the results of the association between HDZH2OH and ethidium bromide (EtBr). EtBr alone exhibited an MIC of 12.69 µg/mL (0.034 mM), whereas its association with the compound reduced the MIC to 8 µg/mL (0.020 mM). However, this reduction was not statistically significant, indicating that HDZH2OH does not appear to exert a relevant modulatory effect on the NorA efflux system in strain 1199B. Since EtBr is a recognized efflux pump substrate, this result provides indirect functional evidence only, and direct confirmation would require dedicated accumulation or efflux assays.

Figure 2C presents the MIC values obtained for ciprofloxacin, used as a specific antibiotic for the MepA pump in strain K2068. In the absence of the compound, the observed MIC was 16 µg/mL (0.048 mM), while in the presence of HDZH2OH, it increased to 25.40 µg/mL (0.080 mM). This behavior suggests the absence of a potentiating effect and may indicate a negative interference with antibiotic activity, possibly due to chemical interactions between the compound and the drug. Figure 2D demonstrates the MIC values of ethidium bromide alone and in association with HDZH2OH and standard inhibitors (CPZ and CCCP). EtBr alone exhibited an MIC of 8 µg/mL (0.02 mM), which was reduced to 2 µg/mL (0.005 mM) in the presence of the compound (*p* < 0.001), indicating a statistically significant reduction. This result suggests that HDZH2OH may interfere with MepA-mediated efflux and supports its potential as an efflux pump modulator in this strain. As with the NorA assays, these findings represent indirect functional evidence, and direct mechanistic confirmation would require fluorometric accumulation or efflux studies.

These findings suggest that HDZH2OH exhibits selective activity, possibly related to structural and functional differences between the NorA and MepA efflux pumps, which, although both belong to the MFS family, present distinct substrate-binding sites and extrusion mechanisms (Schindler et al. 2015; Costa et al. 2022a, b). The lack of intrinsic antibacterial activity of HDZH2OH (MIC ≥ 3.88 mM) can be rationalized by its structural features, since hydrazone derivatives and related phthalazine-based compounds are widely reported to show weak or negligible direct antibacterial effects while acting mainly as resistance modulators rather than bactericidal agents, particularly through interference with bacterial efflux systems (Rollas and Küçükgüzel 2007). Its rigid and highly conjugated framework, composed of a phthalazinyl heteroaromatic core, a hydrazone (C = N) linkage, and an ortho-hydroxylated phenyl ring, together with multiple hydrogen-bond donor and acceptor sites (NH, OH, and imine functions) confirmed by NMR and UV data, supports strong non-covalent interactions and membrane affinity rather than direct lethal activity, which is consistent with its observed ability to modulate antibiotic response, as seen in the partial reduction of norfloxacin MIC against *S. aureus* 1199B via interference with NorA-mediated efflux (Costa et al. 2022a, b). However, the weaker effect compared to classical inhibitors such as CCCP and CPZ suggests only partial modulation of transport activity, while the absence of ciprofloxacin potentiation and the apparent antagonistic effect in *S. aureus* K2068 may be explained by π–π stacking or other non-covalent interactions between the extended aromatic system of HDZH2OH and fluoroquinolones, reducing the effective concentration of the antibiotic, a phenomenon previously described for aromatic nitrogen-containing compounds interacting with quinolones (Tintino et al. 2020; Nascimento et al. 2023). Conversely, the significant reduction in ethidium bromide MIC in the presence of HDZH2OH in strain K2068 supports selective inhibition of the MepA efflux system, reinforcing the concept that structurally conjugated hydrazones may display transporter-dependent and strain-dependent modulation of multidrug resistance rather than broad-spectrum antibacterial activity (Costa et al. 2022a, b).

Molecular modeling and docking studies performed with structurally related hydrazones have demonstrated stable interactions with internal regions of efflux pumps, further supporting this mechanistic hypothesis (Santos et al. 2022; Coutinho et al. 2023). Overall, the results corroborate the literature by demonstrating that the hydralazine-derived hydrazone evaluated in this study, in agreement with previous reports on related hydrazones, acts primarily as a resistance modulator, with effects dependent on the bacterial strain, efflux pump type, and substrate used. The differential activity of HDZH2OH against NorA and MepA highlights the importance of individualized evaluations and reinforces the potential of these compounds as adjuvants in the treatment of multidrug-resistant *S. aureus* infections.

**Fig. 3:**
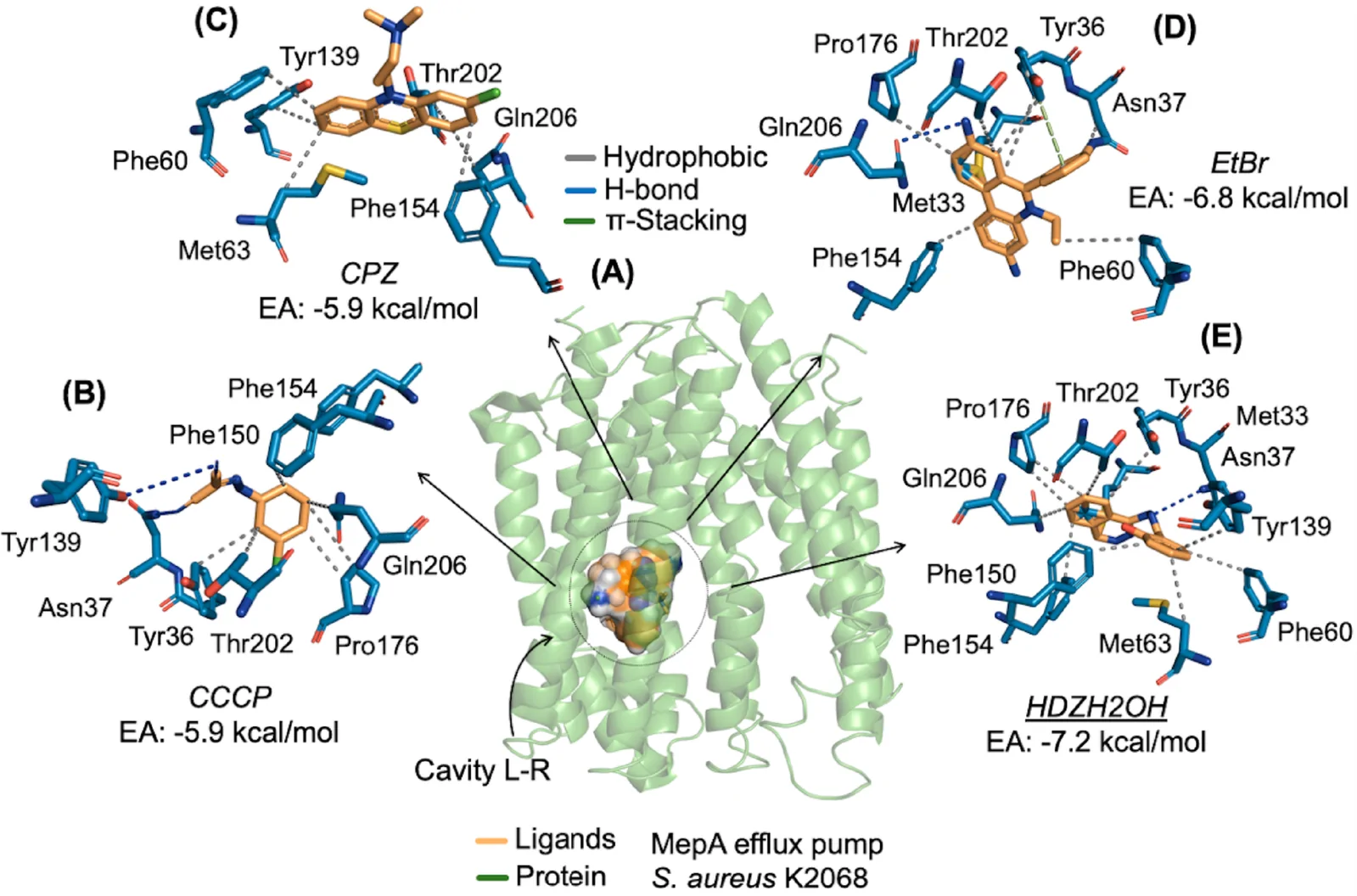
**A** Three-dimensional visualization of ligand docking within the MepA efflux pump of *S. aureus* K2068, showing interactions between the receptor amino acid residues (blue) and ligands (nude). **B–E** Detailed ligand–receptor interaction profiles for CCCP (**B**), CPZ (**C**), EtBr (**D**), and HDZH2OH (**E**)

Based on a set of independent molecular docking simulations, it was observed that the ligand HDZH2OH exhibits interaction affinity for the MepA efflux pump from the *S. aureus* K2068 strain, occupying the same binding domain as the substrates CCCP and EtBr, as well as the control compound chlorpromazine (CPZ) (Fig. 3A). This suggests a relevant spatial overlap that may enable modulation of the protein’s activity. Further analysis of the docking-derived complexes revealed that HDZH2OH shows significant affinity toward MepA, with a calculated binding energy of − 7.2 kcal/mol and an RMSD of 1.997 Å. The ligand also demonstrated high spatial selectivity for the binding site, primarily stabilized by hydrophobic contacts with aliphatic regions and aromatic interactions involving the residues Phe60, Met63, Phe150, Phe154, Thr202, and Gln206 (Fig. 3E; Table 1). These findings indicate a directed binding mode that is strongly dependent on lipophilic complementarity.

**Table 1:** Data on ligand-receptor (L-R) interactions in the redocking process of the agonist CPZ, classic substrates CCCP and EtBr, and molecular docking simulations of the HDZH2O, via MepA efflux pump receptor

Compd	EA (kcal/mol)	RMSD (Å)	Inter. type	Residues/distances (Å)
CCCP	-5.9	1.425	Hydrophobic	Tyr36 (3.94), Phe150 (4.43), Phe154 (3.45), Pro176 (3.56), Pro176 (4.55), Thr202 (3.86), Gln206 (4.49)
			H-bond	Asn37 (4.07), Tyr139 (3.94)
CPZ	-5.9	1.448	Hydrophobic	Phe60 (4.09), Met63 (4.02), Tyr130 (4.47), Phe154 (3.65), Thr202 (4.61), Gln206 (3.90)
EtBr	-6.8	1.524	Hydrophobic	Met33 (0.72), Tyr36 (3.98), Tyr36 (4.93), Asn37 (3.72), Phe60 (4.61), Phe154 (3.56), Pro176 (4.19), Thr202 (4.73)
			H-bond	Gln206 (3.13)
			π-Stacking	Tyr36 (5.33)
HDZH2OH	-7.2	1.997	Hydrophobic	Met33 (0.99), Tyr36 (4.52), Phe60 (4.24), Met63 (4.27), Tyr139 (4.86), Phe150 (3.57), Phe154 (3.90), Pro176 (4.15), Pro176 (3.66), Thr202 (4.76), Gln206 (4.70)
			H-bond	Asn37 (3.16)

CCP: carbonyl-*m*-chlorophenyl hydrazone cyanide. CPZ: Chlorpromazine. EtBr: Ethidium bromide

Ethidium bromide (EtBr; −6.8 kcal/mol, 1.524 Å) and carbonyl cyanide m-chlorophenyl hydrazone (CCCP; −5.9 kcal/mol, 1.425 Å), both classical substrates of the MepA efflux pump, showed interactions within similar regions of the binding domain (Fig. 2B and D), involving the residues Phe154, Thr202, Gln206, Pro176, and Met33, with contact distances ranging from 0.72 to 4.7 Å (Table 1). The control compound chlorpromazine (CPZ) displayed a binding affinity of − 5.9 kcal/mol and an RMSD of 1.448 Å, performing less favorably than the HDZH2OH derivative, which exhibited a more favorable binding energy compared with both the classical substrates and the control (Fig. 3C; Table 1).

Interaction analyses revealed that, although EtBr and CCCP share the same binding domain, their associations are mainly sustained by hydrophobic contacts, with a significant contribution from aromatic π–π stacking interactions, which define the predominant chemical nature of these interactions (Fig. 3B and D). Accordingly, EtBr and CCCP are stabilized predominantly by hydrophobic contacts and π–π stacking, reflecting a more generic and less selective binding mode. In contrast, HDZH2OH not only exhibited higher affinity but also established additional and more directed interactions, suggesting a more stable and selective binding to the MepA site. This profile distinguishes the derivative as a potential competitive modulator, capable of overcoming the limitations of nonspecific recognition observed for classical substrates.

**Fig. 4:**
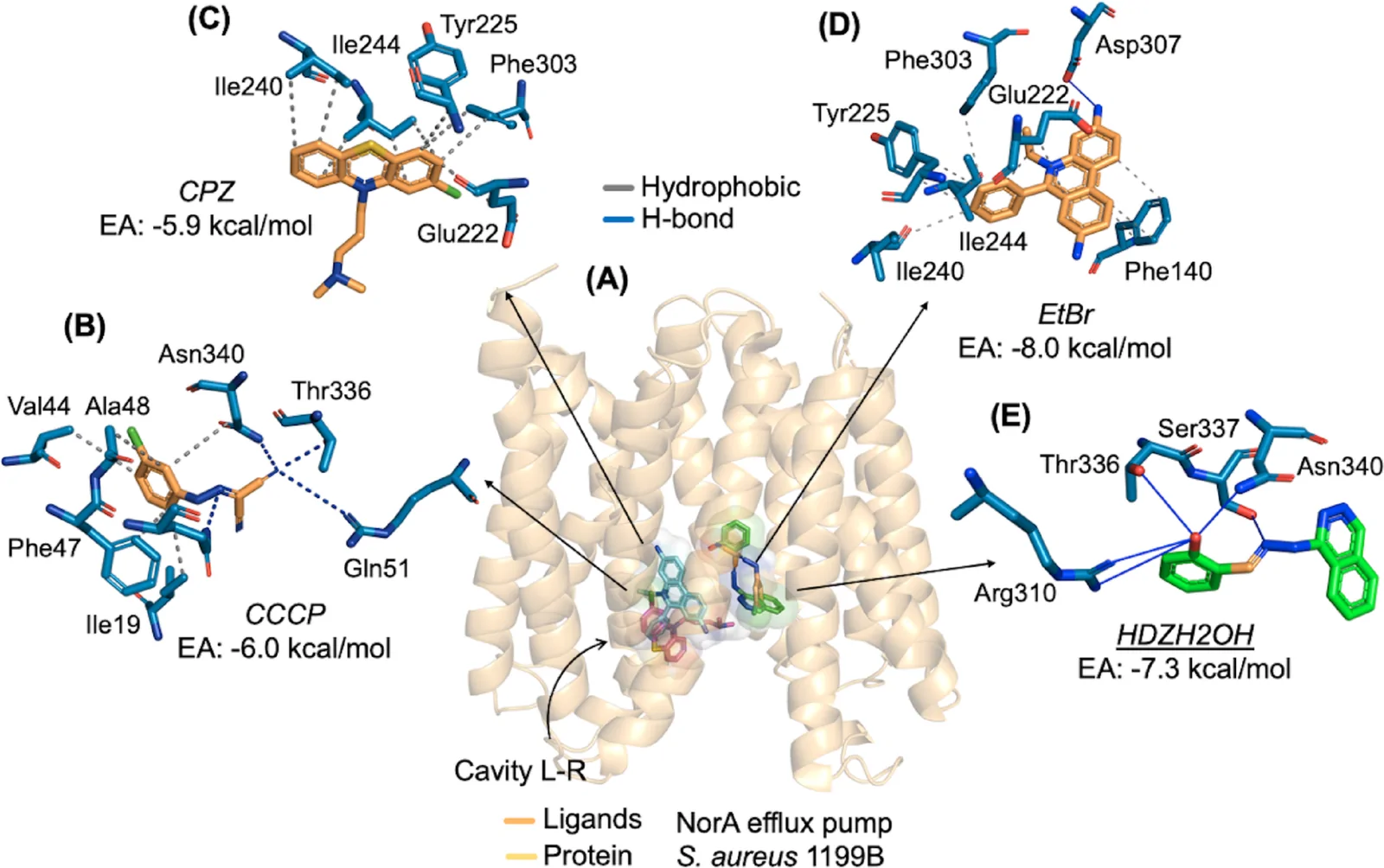
**A** Three-dimensional visualization of ligand docking within the NorA efflux pump of *S. aureus* 1199B, highlighting interactions between amino acid residues (blue) and ligands (nude). **B–E** Ligand–receptor interaction profiles for CCCP (**B**), CPZ (**C**), EtBr (**D**), and HDZH2OH (**E**)

Investigation of molecular docking simulations for the NorA efflux pump from *Staphylococcus aureus* 1199B revealed that HDZH2OH occupies a region adjacent to the binding sites of the substrates CCCP (− 6.0 kcal/mol, 1.927 Å) and CPZ (− 5.9 kcal/mol, 1.693 Å). This spatial difference suggests that, although HDZH2OH does not bind to exactly the same interaction site, it may interfere with the dynamics of the efflux channel, partially competing with these ligands and potentially modulating substrate export (Table 2; Fig. 4A).

**Table 2:** Data on ligand-receptor (L-R) interactions in the redocking process of the agonist CPZ, classic substrates CCCP and EtBr, and molecular docking simulations of the HDZH2O, via NorA efflux pump receptor

Compd	EA (kcal/mol)	RMSD (Å)	Inter. type	Residues/distances (Å)
CCCP	-6.0	1.927	Hydrophobic	Ile19 (3.91), Val44 (4.49), Phe47 (3.78), Ala48 (4.61), Asn340 (4.74)
			H-bond	Gln51 (3.06), Arg310 (3.95), Thr336 (2.21), Asn340 (2.49)
CPZ	-5.9	1.693	Hydrophobic	Glu222 (3.73), Tyr225 (3.74), Ile240 (4.27), Ile240 (3.68), Ile244 (3.90), Ile244 (3.62), Ile244 (3.86), Phe303 (4.35), Phe303 (3.74)
EtBr	-8.0	1.674	Hydrophobic	Phe140 (4.74), Phe140 (3.87), Phe340 (3.83), Glu222 (4.37), Glu222 (4.37), Tyr225 (3.71), Ile240 (4.69), Ile244 (3.48), Phe303 (4.79)
			H-bond	Asp307 (2.83)
HDZH2OH	-7.3	1.442	H-bond	Arg310 (2.97), Arg310 (4.32), Thr336 (3.38), Ser337 (3.80), Asn340 (2.45)

CCCP: carbonyl-*m*-chlorophenyl hydrazone cyanide. CPZ: Chlorpromazine. EtBr: Ethidium bromide

Furthermore, ligand–receptor interaction analyses revealed distinct interaction profiles among the evaluated compounds. CCCP established hydrophobic contacts with the residues Ile19, Val44, Phe47, Ala48, and Asn340, in addition to forming four hydrogen bonds with Gln51, Arg310, Thr336, and Asn340, with interaction distances ranging from 2.2 to 4.7 Å. This suggests a relatively well-directed binding mode and a potentially stabilizing complex (Fig. 4B; Table 2). In contrast, CPZ interacted mainly through hydrophobic contacts with the residues Glu222, Tyr225, Ile240, Ile244, and Phe303, with distances ranging from 3.6 to 4.2 Å (Fig. 4C; Table 2), and without hydrogen bond contributions, reflecting a less specific and potentially more flexible association within the efflux channel.

Nevertheless, the HDZH2OH compound occupies the same binding site as the classical substrate EtBr in the NorA efflux pump of *S. aureus* 1199B. This spatial overlap suggests that HDZH2OH may compete with the natural substrates of the protein, acting as a potential competitive inhibitor. Although the affinity of HDZH2OH (− 7.3 kcal/mol, 1.442 Å) is slightly lower than that observed for EtBr (− 8.0 kcal/mol, 1.674 Å) (Fig. 4D and E), it remains higher than that of CCCP and CPZ (− 5.9 kcal/mol, 1.693 Å), indicating a satisfactory propensity of the ligand to occupy and remain within the active site. Thus, the combination of considerable affinity and overlapping positioning with EtBr supports an inhibition mechanism based on direct competition for the efflux site, with the potential to reduce the export of antibiotics or other substrates and, consequently, restore antimicrobial efficacy against resistant strains. A detailed analysis of ligand–receptor interactions revealed distinct binding profiles for EtBr and HDZH2OH in the NorA efflux pump. EtBr establishes multiple hydrophobic interactions with aromatic and aliphatic residues lining the channel, including Phe140, Phe340, Tyr225, Ile240, Ile244, and Phe303, with distances ranging from 3.48 to 4.79 Å, in addition to forming a hydrogen bond with Asp307 (2.83 Å) (Table 2).

HDZH2OH, in turn, displays a complementary interaction pattern, forming multiple hydrogen bonds with Arg310, Thr336, Ser337, and Asn340 (distances between 2.45 and 4.32 Å), generating a network of polar interactions that promote stability and selectivity within the binding site. Unlike EtBr, HDZH2OH does not rely predominantly on aromatic contacts; instead, its hydrogen-bond network enables strong and potentially competitive binding, occupying regions adjacent to the substrate and suggesting an inhibition mechanism based on partial competition within the efflux channel.

A comparative summary of binding energies and RMSD values obtained from the simulations is provided in Table 3. This overview enables a clear assessment of the relative affinities and conformational stability of the ligand receptor complexes formed with the MepA and NorA efflux pumps of *S. aureus*. The results highlight the performance of the HDZH2OH compared with classical substrates (EtBr and CCCP) and the control compound (CPZ), reinforcing its potential role as a competitive modulator of efflux activity.

**Table 3:** Comparative binding affinities (kcal/mol) and RMSD values of HDZH2OH, EtBr, CCCP, and CPZ against the MepA and NorA efflux pumps of *S. aureus*

Ligand	MepA Binding Energy (kcal/mol)	MepA RMSD (Å)	NorA Binding Energy (kcal/mol)	NorA RMSD (Å)
HDZH2OH	−7.2	1.997	−7.3	1.442
EtBr	−6.8	1.524	−8.0	1.674
CCCP	−5.9	1.425	−6.0	1.927
CPZ	−5.9	1.448	−5.9	1.693

The physicochemical parameters and drug-likeness properties of HDZH2OH are summarized in Table 4 and illustrated in the bioavailability radar shown in Fig. 4B. The molecular weight (264.29 g/mol) and the topological polar surface area (TPSA, 70.40 Å²) indicate that the molecule is compact and sufficiently polar to favor cellular permeability. This profile is consistent with the lipophilicity estimated by partitioning algorithms, with logP values of 3.53 (ChemAxon) and 2.59 (Crippen method), which comply with Lipinski’s rule of five and Veber’s rules for good oral bioavailability.

**Table 4:** Physicochemical properties and drug-likeness profile of the chalcone HDZH2O

Physicochemical property	Value	Source
Molecular weight	264.29	CheAxon
logP	3.53	CheAxon
WlogP	2.59	Wildman method
H-bond acceptors	4.0	CheAxon
H-bond donors	2.0	CheAxon
Rotatable bonds	3.0	CheAxon
Topological Polar Surface Area	70.40	CheAxon
pKa (most basic center)	4.05	CheAxon
logD at pH 6.4	3.53	CheAxon
logD at pH 7.4	3.53	CheAxon
logS	-4.44	CheAxon
Rule of five	Accepted	Lipinski method
GSK rule	Accepted	Veber method
CNS MPO score (Pfizer)	4.47	CheAxon

**Fig. 5:**
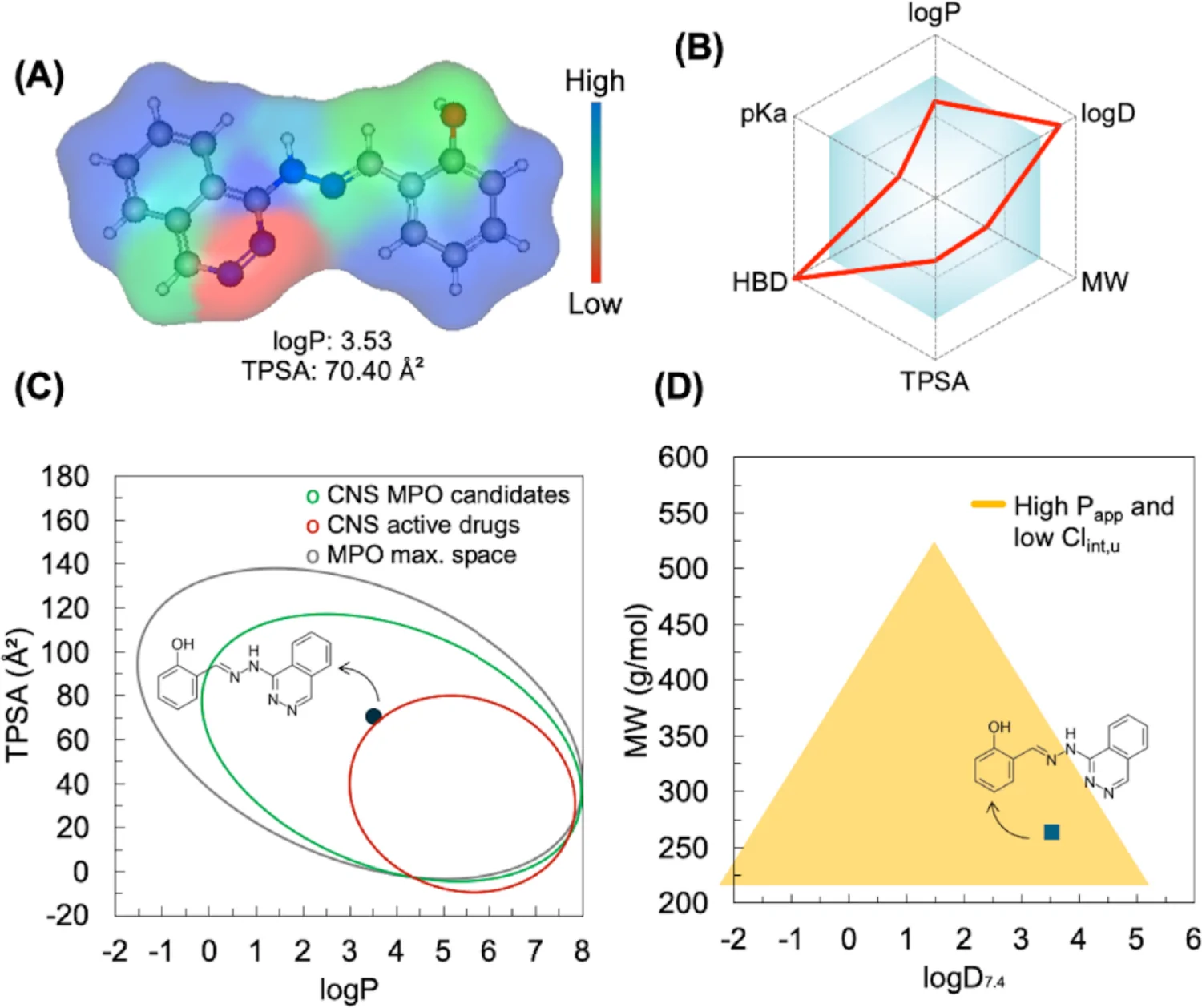
**A** Surface map of molecular lipophilicity potential (MLP) for the base conjugated to HDZH2OH (logD7.4). **B** Multiparameter optimization (MPO) radar illustrating ADME desirability. The MPO profile was further evaluated by integrating: **C** topological polar surface area (TPSA) and logP to estimate CNS permeability, and **D** molecular weight (MW) and logD at pH 7.4 to assess oral absorption and metabolic stability

The pH-dependent lipophilicity is detailed in the graph shown in Fig. 5A and in the data presented in Table 4. The pKa value of 4.05, associated with an amine group present in one of the aromatic rings, indicates a basic center capable of modulating lipophilicity under physiological conditions. The similarity between logD and logP values, both close to 3.53, suggests that the molecule predominantly exists in its neutral form, exhibiting an adequate balance between lipophilic and hydrophilic properties, a feature favorable for intestinal permeability and absorption.

Application of the CNS Multiparameter Optimization (CNS MPO) index, shown in Fig. 5C, yielded a score of 4.47, indicating that the molecule meets drug-likeness criteria and presents desirable attributes such as high passive permeability (Papp), low P-glycoprotein (P-gp)–mediated efflux, and reduced metabolic clearance (Yee 1997). These parameters place HDZH2OH within the quadrant of CNS-active compounds in the Pfizer reference set, as illustrated in Fig. 5D, reinforcing its promising profile for central nervous system (CNS)-related applications. The physicochemical characteristics of HDZH2OH are directly related to its predicted bioavailability, estimated as a 55% probability of achieving at least 10% oral absorption in rat models, which are commonly used as predictors for human absorption. As a molecule that is neutral under physiological conditions and compliant with Lipinski’s rule of five, HDZH2OH shows a low probability of P-glycoprotein (P-gp) transport, favoring high human intestinal absorption (HIA = 93.57%) (Daina et al. 2017) (Table 5).

**Table 5:** Pharmacokinetics by the ADME models of the SwissADME and preADMET web servers

ADME profile	PreADMET	SwissADME
F > 10% (Bioavailability)	Not reported	0.55
P-gp inhibitor	No	Not reported
P-gp substrate	Not reported	No
HIA (Human intestinal absorption)	93.57%	High
PPB (Plasma protein binding)	92.36%	Not reported
BBB (Blood–Brain Barrier)	5.53 × 10⁻^5^ cm/s (Papp)*	+++
CYP3A4 substrate	No	Not reported
CYP2D6 substrate	No	Not reported
CYP3A4 inhibitor	No	No
CYP2D6 inhibitor	No	No
CYP2C9 inhibitor	Yes	No
CYP2C19 inhibitor	Yes	No

BBB penetration of the PreADMET server estimated by the in vitro passive permeability (Papp) model of the Madin-Darby Canine Kidney (MDCK) cell line

The estimated plasma protein binding (PPB = 92.36%) suggests extensive distribution between plasma and peripheral tissues, potentially increasing the likelihood of CNS exposure. Additionally, the calculated apparent permeability (Papp = 5.53 × 10⁻⁵ cm/s) in the in vitro Madin–Darby canine kidney (MDCK) cell model, obtained using PreADMET, supports the in silico prediction of good blood–brain barrier (BBB) permeability according to the SwissADME BOILED-Egg model (Table 5). Metabolic profiling of HDZH2OH with respect to cytochrome P450 (CYP450) isoforms indicates a low propensity for metabolism by CYP3A4 and CYP2D6, suggesting reduced clearance and a potentially prolonged plasma half-life. In parallel, HDZH2OH shows a strong potential to inhibit CYP2C19 and CYP2C9 isoforms, which may lead to increased plasma concentrations of coadministered drugs that depend on these enzymes for biotransformation (Table 5).

**Fig. 6:**
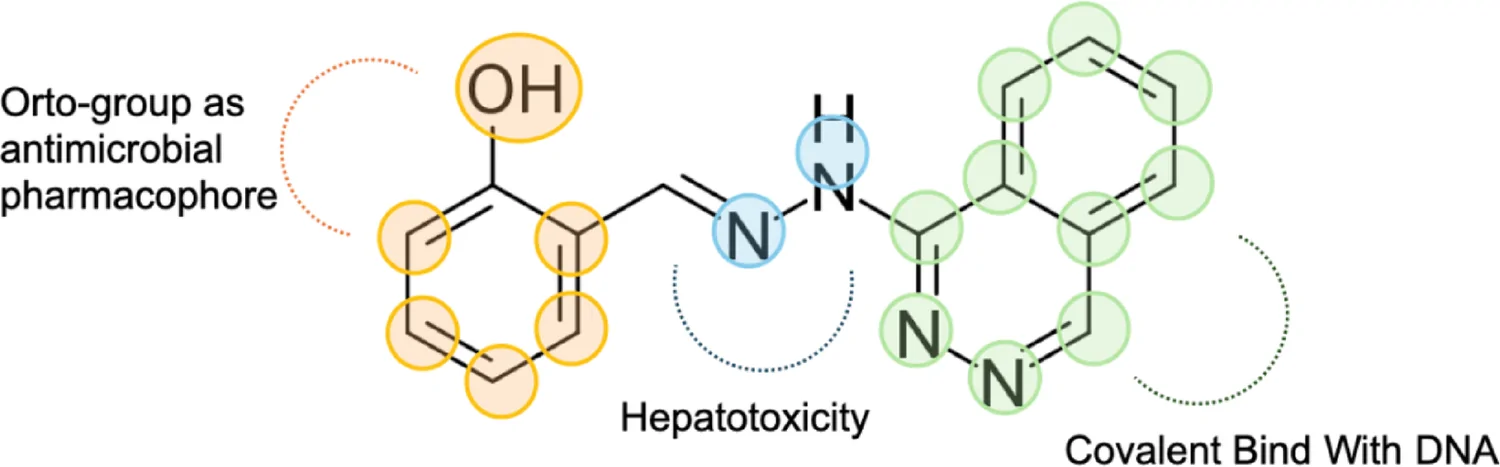
Structural contributions and pharmacophores

The toxicological assessment of HDZH2OH, performed using the PreADMET and ProTox-II platforms as well as eMolTox substructure screening, suggests that the aniline fragment may be metabolically unstable and could contribute to hepatotoxicity (drug-induced liver injury, DILI). Additionally, the phthalazine fragments show potential for covalent interaction with DNA, indicating a possible mutagenic risk; however, the ortho-substituted group on the benzene ring may enhance selectivity toward antimicrobial activity (Fig. 6). Nevertheless, the predicted lethal dose (LD₅₀ = 350 mg/kg) indicates that adverse effects may occur at relatively high doses, suggesting a moderate toxicological risk under normal physiological exposure conditions (Table 6).

**Table 6:** Toxicity end-points and oral lethal dose (LD50) by the consensual prediction of PreADMET and ProTox-II web-tools

Toxic property	PreADMET	ProTox-3.0
DILI	Not reported	+ (0.62)
AMES Mutagenic	Mutagenic	+ (0.74)
hERG inhibitor	Low risk	Not reported
LD50	Not reported	350 mg/kg (class 4)

The results indicating that the HDZH2OH does not possess intrinsic antibacterial activity MIC values ≥ 1024 µg/mL (3.88 mM) but instead functions primarily as a selective efflux pump modulator with strain-dependent effects. In microbiological assays, HDZH2OH showed limited potentiation of norfloxacin against the NorA overexpressing strain 1199B, while displaying a significant reduction in ethidium bromide MIC against the MepA expressing strain K2068, suggesting stronger modulation of the MepA system but inconsistent effects across antibiotics and targets. These findings are mechanistically supported by docking simulations, which reveal favorable binding of HDZH2OH to MepA (− 7.2 kcal/mol) within the substrate-binding region shared with classical ligands such as EtBr and CCCP, stabilized by hydrophobic and aromatic interactions with key residues, whereas in NorA the compound binds an adjacent region and forms a hydrogen bond network that may lead to weaker or indirect interference with efflux activity. This differential binding behavior explains the experimental selectivity observed between the two efflux systems.

Complementarily, ADME predictions indicate that HDZH2OH possesses physicochemical properties compatible with membrane permeability and intracellular access, including moderate lipophilicity, acceptable molecular weight, and suitable polar surface area, supporting its ability to reach efflux pump targets, while also suggesting favorable oral absorption and potential CNS permeability; however, predicted CYP450 inhibition and moderate toxicity risks (including possible hepatotoxicity and mutagenicity associated with structural fragments) highlight pharmacokinetic and safety limitations. Altogether, the convergence of experimental and computational data demonstrates that HDZH2OH acts as a membrane-permeable, selectively binding efflux pump modulator with greater efficacy toward MepA than NorA, and its overall activity is governed by a balance between structural compatibility with efflux transporters and pharmacokinetic constraints that will be critical for future optimization.

## Conclusion

HDZH2OH exhibited a cytotoxic effect on macrophages, significantly reducing cell viability in a concentration-dependent manner. HDZH2OH did not exhibit intrinsic antibacterial activity against *S. aureus* strains overexpressing the NorA and MepA efflux pumps, but it demonstrated clear efflux modulating properties, with selective interference of the MepA system in strain K2068 as shown by a significant reduction in ethidium bromide MIC. In contrast, only partial and substrate-dependent potentiation of norfloxacin was observed in the NorA-overexpressing strain 1199B, while the lack of effect on ethidium bromide further supports a selective and contexto dependent mechanism of action. Collectively, these results identify HDZH2OH as a novel hydrazone scaffold with preferential activity as an efflux resistance modulator rather than a direct antibacterial agent, supported by complementary docking and in vitro findings indicating differential interactions with efflux systems. Although pharmacokinetic predictions suggest favorable drug-like properties, these remain preliminary and require experimental validation. Overall, the study highlights the potential of HDZH2OH as a lead structure for efflux pump modulation strategies, while its therapeutic relevance remains speculative at this stage; future work should prioritize mechanistic confirmation of efflux inhibition, evaluation against clinically relevant multidrug-resistant isolates, structural optimization to improve potency and selectivity, and in vivo studies to establish pharmacological efficacy and safety.

## Supplementary Information

Below is the link to the electronic supplementary material.


Supplementary Material 1


## Data Availability

No datasets were generated or analysed during the current study.
