# Supplementary material for: Evaluation of the antimicrobial and NorA and MepA efflux pump inhibitory activity of a hydrazone derivative of hydralazine against Staphylococcus aureus
Source: Arch Microbiol. 2026 Jun 19;208(9):446. doi: 10.1007/s00203-026-05007-0 (PMC13282286; doi:10.1007/s00203-026-05007-0)
Supplement: Supplementary file 1 — Supplementary Material 1 [file 203_2026_5007_MOESM1_ESM.docx]

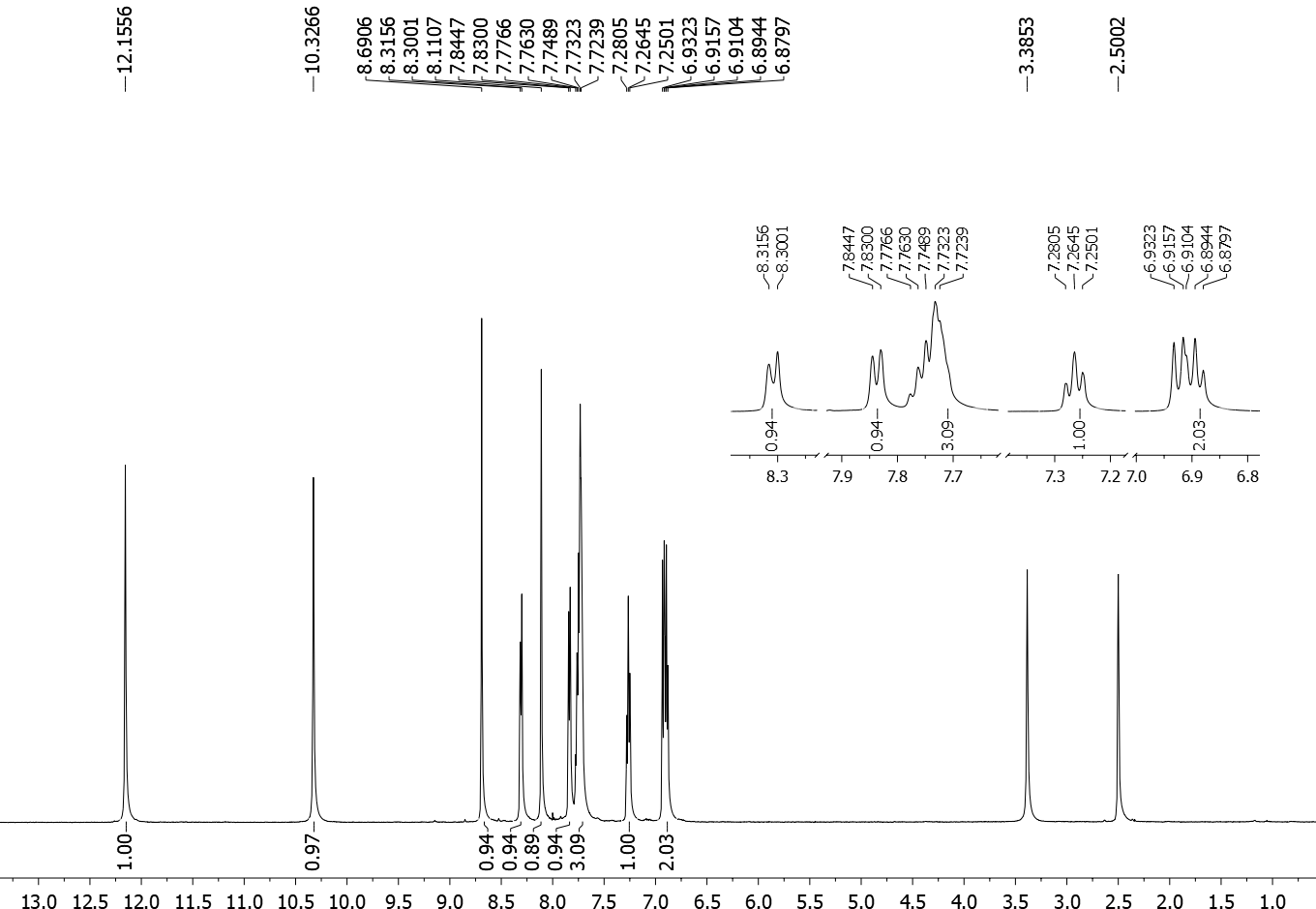


**Figure S1.** ^1^H NMR (500 MHz, in DMSO) spectrum of compound


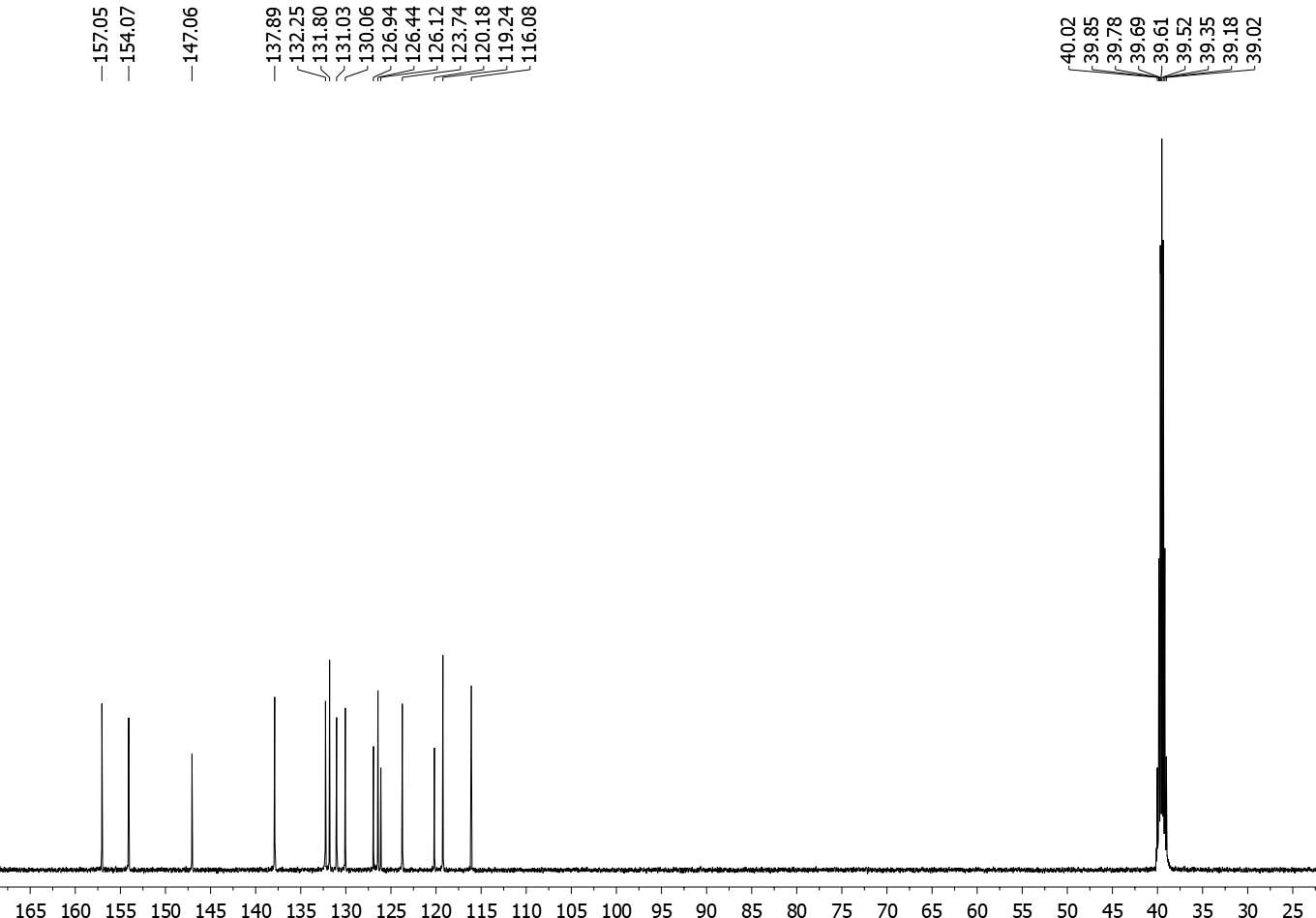


**Figure S2.** ^13^C NMR (125 MHz, in DMSO) spectrum of compound

| **Description** | **Retention Time (min)** | **%** |
| --- | --- | --- |
| Peak | 3.512 | 99.6926 |

**Figure S3.** HPLC chromatogram of the compound

| **Description** | **Maximum wavelength (nm)** |
| --- | --- |
| Peak | 210; 287; 370 |

**Figure S4.** UV–Vis Spectrum of the compound
